# Personal

**Published:** 1893-05

**Authors:** 


### Personal.

DR. T. F. DWYER has moved to 89 West avenue.

DR. F. W. ABBOTT has moved to 523 Franklin street.

DR. HERMAN MYNTER has moved to 566 Delaware avenue.

DR. WILLIAM H. PLEUTHNER has returned from his European trip.

DR. W. S. TREMAINE has returned from an extended trip abroad.

DR. W. H. HEATH has moved to the corner of Allen and North Pearl streets.

DR. WILLIAM H. BERGTOLD has been appointed bacteriologist to the Health Department.

DR. ELMER STARR, whose practice is confined to diseases of the eye and ear, has removed his office to 412 Franklin street, near Virginia. Hours, 9 A. M. to 1 P. M.

DR. VANDERVEER, of Albany, has been nominated by the republican caucus to fill a vacancy in the Board of Regents. A better selection could not have been made, and we trust that the democrats will see the wisdom of this choice and make his election unanimous.

